# The magnitude and importance of perceived health dimensions define effective tailor-made health-promoting interventions per targeted socioeconomic group

**DOI:** 10.3389/fpubh.2022.849013

**Published:** 2022-10-17

**Authors:** John A. J. Dierx, Hans D. P. Kasper

**Affiliations:** ^1^Department of Caring Society, Research Group Living in Motion, Avans University of Applied Science, Breda, Netherlands; ^2^Department of Marketing and Market Research, Maastricht University School of Business and Economics, Maastricht University, Maastricht, Netherlands

**Keywords:** positive health, socioeconomic status, self-rated health, subjective approach, Netherlands, tailored interventions

## Abstract

Recent insights and developments on health and society urge a critical look at the positive relationship between socioeconomic status (SES) and health. We challenge the notions that it is sufficient to distinguish only between two groups of SES (low and high) and that only overall health is taken into account. A new grouping of SES was developed based on both income and education, resulting in six SES groups. Health was defined in terms of a new positive health concept, operationalized into six health dimensions generating a measure of total general health (TGH). Next, six socioeconomic and demographic determinants of health were included. Linear regression, *T*-tests and one-way ANOVA were applied to investigate the relationships in a Dutch sample. A subjective way to measure health was applied: self-rated health (SRH). As a result, four out of six dimensions of health determined TGH: bodily functions, daily functioning, quality of life, and social and societal participation. Three out of six socioeconomic and demographic determinants impacted TGH: housing situation, age, and difficulties meeting financial obligations. While this is the general picture for the entire sample, there were interesting similarities and differences between the six SES groups. The similarities lie in the positive impact of the evaluation of bodily functions and daily functioning on TGH in all SES groups. The other dimensions affected TGH in some groups, and some dimensions only in one SES group. None of the socioeconomic and demographic determinants affected TGH in all SES groups. New insights on health inequalities are provided. It is concluded, first that the well-known positive relationship between SES and health is confirmed in this study. Second, further refining the health concept into six dimensions provides more detailed insights on which dimensions impact health the most. The subjective approach applied offers more refined information to better understand which health issues really matter to people. This yields new insights to develop tailor-made interventions aimed at increasing healthy behaviour in specific societal groups.

## Introduction

The impact of people's socioeconomic status (SES) on health has been studied extensively (1–3). An elaborate literature review by Petrovic et al. (3) shows that such studies differ in four aspects. First, outcome parameters range from biological indicators (4–6) to health behaviour parameters (7–9), morbidities like cardiovascular disease and diabetes (10–14) and all-cause mortality (8, 9, 15). Second, SES is constituted in a non-uniform way with most studies using gross household income, parental education and employment (or occupation) as SES parameters (4, 9, 13, 15, 16). Third, in almost all studies the effect of each SES parameter on the outcome parameter is studied separately, where some dichotomise the multilevel SES parameters into high and low SES (17). Only a few create an overall SES parameter based on income, education and/or employment (or occupation) (9, 18, 19). Fourth, studies differ in measured objective parameters of health and SES vs. subjective or self-rated health (5-level score, range poor-to-good) and SES (9, 17–20). In this study we investigate a multidimensional view on both SES and self-rated health (SRH) by distinguishing six groups of SES and six dimensions of health next to Total General Health (TGH) to gain more refined insights into the complex relationship between SES and health. Our study is unique in three ways. We:

- Construct a SES variable based on the respondents' individual combined score on their household income as well as their highest educational level;
- Apply a six-dimensional health concept based on the presence of people's health instead of their absence of health as measured by illness or disease in terms of mortality of morbidity;
- Use people's self-rated (subjective) health instead of objective indicators of health.

SES is an undisputedly major factor influencing TGH and health behaviour. Simply stated, it can be concluded that a positive relationship exists between SES and health (1, 3, 10, 19, 21–23). The socioeconomic determinants of TGH as mentioned and described in the model of Lalonde (24) have been studied elaborately (2, 21, 25–29). The complexity of the relationships between socioeconomic determinants of health has been conceptualised in the rainbow model of Whitehead and Dahlgren (30). This model, implicitly or explicitly, includes age, gender, marital status, household size and employment as socioeconomic determinants of health and depicts the interactions between them at the level of individual lifestyle, social and community network, and general socioeconomic, cultural and environmental conditions. The rainbow model is still the most abundant and complete to date. Many studies describe the socioeconomic health disparities between people with low and high SES scores. Since synonyms like “health inequity” and “health inequalities” are used in the literature for the term “health disparities,” it is important that we use “health inequalities,” following the approach of McCartney et al. (31) (see Socioeconomic determinants of health section).

In light of recent insights and developments on health and society, a critical look at the way SES and health are defined and/or operationalized is needed to expand understanding of their relationships and of interventions to increase healthy behaviour and reduce health inequalities. We will reveal that distinguishing more SES groups on an aggregated level based on education and income and that applying the concept of positive health and its six dimensions will provide these better and more refined insights. This study therefore addresses the following three research questions:

1. What health dimensions determine the perception of TGH?
2. What socioeconomic and demographic variables determine the perception of TGH?
3. To what extent do these relationships differ between various groups of SES distinguishing six instead of two groups of high and low SES?

The implications of the results on future research and on possible health-promoting interventions toward diminishing socioeconomic health inequality are discussed. The empirical study was conducted in the Netherlands.

### Socioeconomic determinants of health

In their overview article, McCartney et al. (31) define health inequalities as “…the systematic, avoidable and unfair differences in health outcomes that can be observed between populations, between social groups within the same population or as a gradient across a population ranked by social position….” We will focus on such systematic and avoidable differences in health outcomes.

In the Netherlands, people from low SES groups report on average living 18 more years with illness and dying 7 years earlier than people from high SES groups (32). There are multiple factors that account for this inequality. In terms of behaviour, people from low SES groups tend to participate more often in risky health behaviours such as smoking, higher fat and lower fruit and vegetable consumption, low physical activity and higher alcohol consumption more often (3, 33–36). This tends to be more pronounced in men than in women, leading ultimately to men reporting lower self-rated health (33, 34) and suffering significantly more from chronic diseases (35). Premature deaths in non-married persons tend to be higher than in married persons (21). Another common factor is unemployment or poor job satisfaction having detrimental effects on health (36–38). Household size has likewise been shown to impact health (39–44). These studies refer to long-term detrimental effects of family SES on health, in the sense of low family income and parents as well as grandparents belonging to low SES groups (44–46). In larger families, mothers tend to stay at home caring for children, so these families have lower income than families where the mother is employed (41–43, 45, 46). By contrast, obesity in siblings living in larger households appears to be less common and is dependent on order of birth, as younger siblings tend to show higher odds of becoming obese (40, 42). Hence these studies suggest a socially hereditary component of SES with family size too as a possible determinant for poor health. Whether and how people can escape from such a socially inherited inequality cage or will remain unhealthy from one generation to the next is a point of discussion, as these inequalities persist for many years and even tend to increase despite interventions to change such situations (44). Crises like the recession of the early 2000 s and the current COVID-19 pandemic might even enhance existing inequalities (43, 47, 48). Petrovic et al. (3) (p. 23) show that “socially disadvantaged individuals tend to adhere more to health detrimental behaviors either due to material and financial constraints, perceptions of fewer benefits of health behaviours for longevity, a lack of knowledge of their detrimental effect, difficulties to take up health promoting messages as well as more pessimistic attitudes about life. Often, the deprived neighbourhoods where they live offer little opportunities for a healthy life.” These health inequalities based on socioeconomic and demographic variables as well as on behaviour seem to be systematic, and whether they can be avoided or changed by specific single health or lifestyle interventions remains debatable. A more multidisciplinary approach is advocated to develop effective interventions (49).

### Socioeconomic status: The concept and its operationalization

The concept of socioeconomic status usually refers to the description of groups of people and the differences in relation to their social class and financial situation. As reviewed by Petrovic et al. (3), SES is operationalised by the determinants of education and income. Two approaches can be distinguished: one applying only one of these two determinants, another a combination of the two. In the latter approach researchers form two groups of people according to their SES by distinguishing between people with low education and low income (= low SES) vs. those with high education and high income (= high SES) (18, 33, 50). We suggest that the relationship between income and education may not be that straightforward at the individual or group level. More recently, Hoes et al. (51) used nine levels of education which were transformed into three levels in the analysis: low, intermediate and high. They also categorised net household income per month into three levels: low, intermediate and high. Separate analyses were performed for the three educational groups as well as for the three income groups. For both income and education, Hoes et al. (51) compared respondents from the high group to those from the low group, and respondents from the intermediate group with those from the low group. However, either no comparison was made between respondents with high income but a low education (or vice versa) or it was not taken into account. In their California Health Interview Survey, Meyer et al. (19) (p 1735) take a next step in developing their SES variable based on respondents' actual level of education and income. They describe their procedure to develop a composite index of SES: “Individuals reported on their education by selecting 1 of 11 options ranging from having no formal education to having a PhD or equivalent. We computed income by dividing total household income by the number of adults residing in the household. We then standardized this value and averaged it with a standardized version of the education variable to create a composite index of SES.” In our study, we operationalised SES more or less similarly by combining the 7-level education variable with a 7-level income variable.

As suggested by Flinterman et al. (52), several participants had low education but were successful high-income entrepreneurs. On the other hand, several participants with high education ended up earning low incomes due to unemployment or negative life events such as divorce or illness. Clearly more combinations of income and education exist than merely the two of low income/low education vs. high income/high education. Recent studies also report the COVID-19 pandemic affecting the often-highly-educated and mid-to-high-income self-employed, rendering them low-income (43, 47, 48). This shows that income can fluctuate during life and is not a fixed given (47). Hence where the traditional and often continuous or dichotomised classification probably discloses the general effects of SES on health, a classification into more SES groups as a combined measure based on the SES determinants of education and income might shed additional and more detailed light on the way SES impacts health. We propose distinguishing six groups of various combinations of income and education.

### Health: The concept and its operationalisation

In 1948, the WHO defined health as “A state of complete physical, mental and social wellbeing and not merely the absence of disease or infirmity” (53). Since then, thanks to developments in society, medicine, science, public health and technology, people's views on health have changed. This has already been elaborately described by others, and several attempts have been made to update this definition to one more suitable to the times (31, 54–57). The main discourse is about including parameters other than health, doing more justice to the individual perception of health, the impact of the physical environment on health, and the fact that “health” is not static but rather dynamic. Elaborating on the original WHO definition adhering to Nordenfelt's conception of health (55, 56) Bircher defined health as “a dynamic state of wellbeing characterized by a physical, mental and social potential, which satisfies the demands of a life commensurate with age, culture, and personal responsibility. If the potential is insufficient to satisfy these demands the state is disease”.

When evaluating this definition we believe it holds an objective professionals' perspective, which is often deficit-based as medical professionals tend to focus is on “what (potential) is lacking” and strive for maximum achievable outcome. Huber et al. (58) took all of this into account and proposed a more “positive” definition of health, with health as “…the ability to adapt and self-manage, in the face of social, physical and emotional challenges.” Such an approach, also criticised for being too general and hence not specific enough (59), holds a subjective patient-centred perspective and is asset-based, focusing on meaningful possibilities striving for optimal outcome. In this respect, the term “assets” can be defined as “any factor (or resource) which enhances the ability of individuals, groups, communities, populations, social systems and/or institutions to maintain and sustain health and wellbeing and to help to reduce health inequities” (60). We would like to emphasise that this positive approach to health focuses on the potential people have instead of what they lack. In light of equality, this means that everyone should be able to have equal opportunity and access to these assets, as they are at the core of the capability approach (54, 61–63).

Huber subsequently operationalised this definition with the concept of positive health, this time adding the three dimensions of meaningfulness, quality of life and daily functioning to bodily functions, mental functions, and social and societal participation (the equivalent of the three WHO dimensions of health) (64). The positive health approach relies on people's own perceived (subjective) evaluation of their health instead of on the professional's (more objective) judgement of a patient's health. In investigating the perceived health of a population, this concept of positive health appears to have added value (65). Traditionally, studies on health tend to use quantitative and rather objective measures, like blood pressure, weight, body mass index and specific diseases (4–6, 12, 13, 23). From the literature on consumer behaviour it is well-documented that not only objective information affects people's behaviour but that behaviour is much more affected by the way people perceive those issues subjectively (66, 67). For example, whether they perceive their illness as serious or whether they perceive their smoking habit as pleasant can determine behaviour to a larger extent than mere factual information on, e.g., the dangers of smoking. In line with this way of thinking, we will apply a subjective approach toward evaluating participants' own health instead of objective factual judgements made by professionals. In line with recent findings (20, 68), we argue that interventions based on such a subjective approach of self-rated health (SRH) might be more effective than interventions based on an objective, professional judgement-based approach. Here using the positive health concept offers the opportunity to focus the interventions on people's own judgement of their health potential (what they can do) instead of their perceived shortcomings (what they cannot do).

## Methods

### Research model

**Figure 1:**
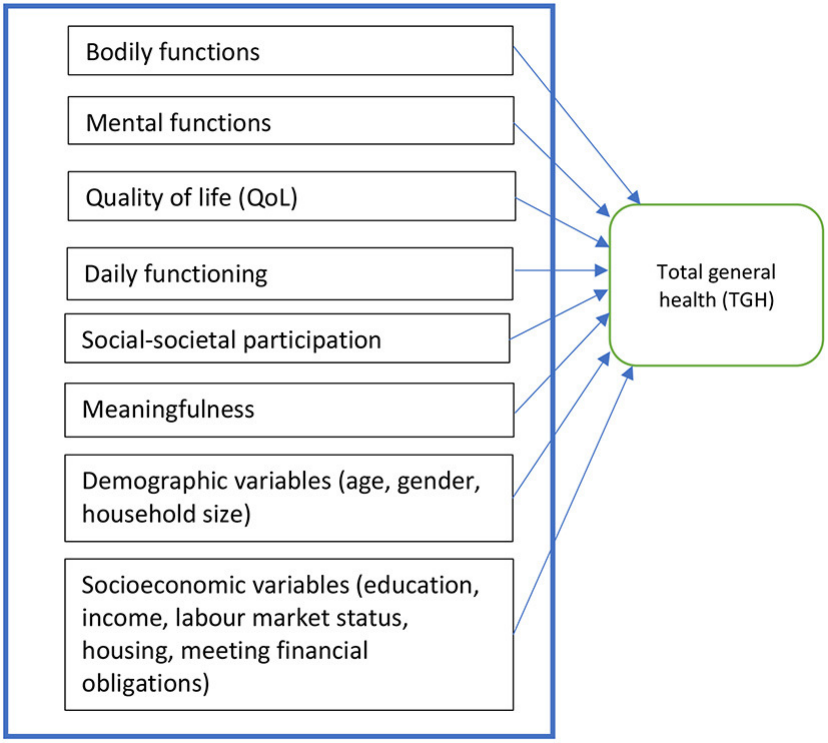
Research model.

The research model as shown in Figure 1 was used to answer the three research questions. We studied whether and how the six dimensions of the positive health concept and several socioeconomic and demographic variables like age, gender, educational level, labour market status, income, household size and housing situation determined perceived TGH in the average Dutch population. We also included the way people perceive their income situation (ability to meet financial obligations) instead of only looking at the amount of gross household income as an explanatory variable in our models.

### Research population and data collection

In the fall of 2016 an online questionnaire was sent to all internet panel participants of RMI, a Dutch commercial market research company. This panel consists of 30,000 respondents aged 18 years and older, and is a representative sample of age, gender and urban-rural population. The respondents are members of the RMI Internet panel and participated in the study voluntarily. At the start of this Internet panel years ago, RMI obtained informed consent from all panel members agreeing to participate in this study panel. To encourage people to participate in studies, respondents receive a small fee of *e*1 for every completed questionnaire.

The 32-item Positive Health questionnaire as developed and kindly made available by Dr Huber (64) was used with some slight adaptations. Other topics were added to measure socioeconomic and demographic status. The final version consisted of 20 questions and 45 items. Participants were asked to assess their total perceived health and their perceived health on each of the six dimensions of the positive health concept after scoring the 32 items of the positive health scale from 1 (poor) to 5 (excellent). In this way all respondents evaluated their health on the same topics, avoiding different interpretations of health (69). The research design opted for analysing the dataset with regression analysis.

Data collection was terminated after a representative sample of 1,000 participants had responded. This number was decided upon because it allows for breaking down into several subgroups if necessary. For instance, if it were interesting to define four or six SES groups, group sizes would still be sufficient for adequate statistical analysis. Ideally, for regression analyses to be performed the number of respondents should be at least the quadrate of the number of independent variables, so with 12 independent variables the number of respondents in a group should ideally be at least 12^*^12 = 144. Since regression analysis is a very robust technique, a lower number of respondents should still allow for proper analysis.

After checking the response for missing data on the SES items income and education, a total of 772 respondents were included in the statistical analysis. Only on educational level did the sample appear not to be representative of the Dutch population. Educational level was slightly higher in the sample compared to the average Dutch population and was corrected for during statistical analysis of the results.

### Ethics

Since participants in this study did not undergo any physical examination and bodily fluids or other medical data were not collected, the Medical Research Involving Human Subjects Act does not apply to the current study. Although this approval does not appear to be mandatory, the Ethics Committee of the Silverbrains Board approved the research design and protocols for the data collection and analysis. The study was conducted in accordance with the rules and guidelines of the Dutch Expertise Center for Marketing Insights, Research and Analytics (MOA) and the Association for Policy Research (VBO), which are in line with the rules and guidelines of the European Society for Opinion and Marketing Research (ESOMAR).

### Statistical analysis

Data of the 772 respondents were analysed using IBM SPSS Statistics 26. Spearman correlations and linear regression analysis were performed, determining the direct relationships and relative contributions of the health determinants as well as the socioeconomic and demographic determinants to perceived TGH. Linear regression is a commonly used statistical technique and a justified way to analyse these relationships (69). *T*-tests and one-way ANOVA were used to test for statistical differences between the SES groups.

The linear regression had TGH as the dependent variable to be explained by several independent variables. In the first regression for the whole sample these independent variables were the six dimensions of health as well as the SES position and the six socioeconomic and demographic variables.

TGH = α + β_1_ Bodily functions + β_2_ Daily functioning + β_3_ Quality of life + β_4_ Social and societal participation + β_5_ Mental functions and perception + β_6_ Meaningfulness + β_7_ SES + β_8_ Home ownership + β_9_ Age + β_10_ Difficulties in meeting financial obligations + β_11_ Household size + β_12_ Labour market status + β_13_ Gender + ε.

Basically, in the regression equation α represents the “constant,” β represents the regression coefficient whose magnitude/size can be considered as an indicator of how important that particular variable is in explaining the dependent variable TGH, and ε represents the error term in such equations.

In the second instance we ran the regression again for the whole sample but without the SES variable, which turned out to be non-significant, rendering twelve independent variables explaining TGH. That model was also run for each subgroup of SES distinguished in our further analyses.

**Figure 2:**
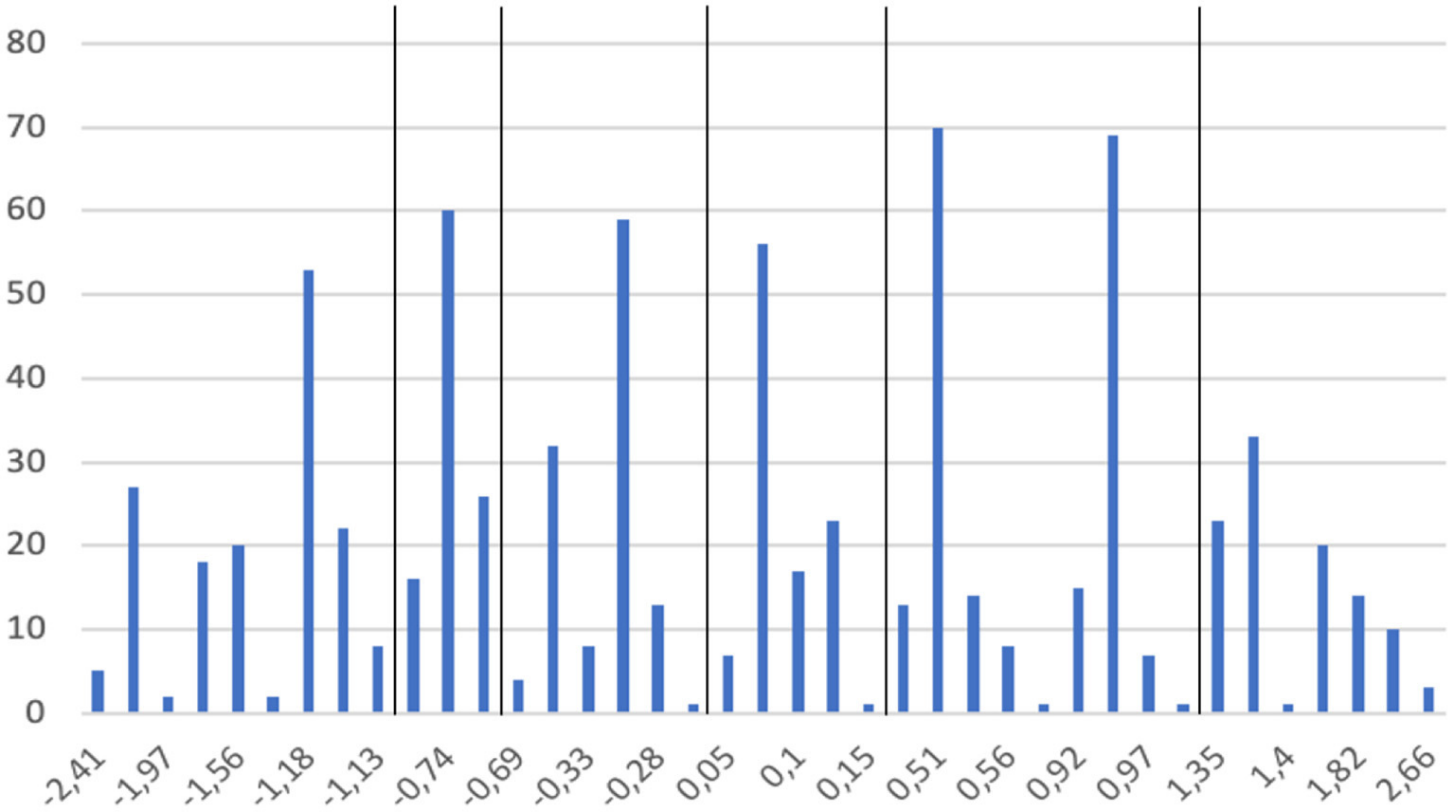
Distribution of individual SES scores.

The evaluation of TGH and each health dimension was given on a five-point Likert scale ranging from 1 = poor to 2 = moderate, 3 = reasonable, 4 = good and 5 = excellent. Home ownership is a dummy variable where 1 = home ownership and 2 = rented home. Age is the actual biological age. Difficulties in meeting financial obligations was measured on a five-point Likert scale ranging from 1 = a lot of difficulties to 2 = difficulties, 3 = some difficulties, 4 = hardly any difficulties and 5 = no difficulties at all. Household size was measured as the total number of persons in the household. Labour market status was measured *via* various positions in the labour market (see **Table 2**); in the regression it is a dummy variable of not having a job (=1) or having a job (=2). Individual SES scores were determined on the basis of respondents' highest achieved educational level (7 levels) and gross household income (7 levels). The procedure to calculate these individual SES scores was as follows:

Individual respondents' SES scores were calculated based on their answers to the two questions on their highest achieved educational level and gross household income (both variables were measured by ticking the appropriate answer on a seven-point scale; see also Table 1). The composite score was calculated *via* the factor analysis module in SPSS to create a new variable for the individual's SES score. In this calculation the mean score of the newly calculated SES variable of all respondents was set at zero, resulting in respondents with an individual SES score below zero while others had an individual SES score above zero (see Figure 2). Figure 2 shows that more groups of respondents can be distinguished than just the two with SES scores below or above zero. In forming these groups, we considered the distances between individual SES scores (e.g., when a “big break” occurred) and the number of respondents in each SES group, as this number should facilitate our regression analysis (meeting the rule that the number of respondents should be larger than the squared number of independent variables in our regression equations).

**Table 1:** Sample and SES groups' composition based on education and income.

		**Two SES groups**		**Six SES groups**					
	**Total sample**	**Low SES**	**High SES**	**Very low SES**	**Low SES**	**Mid-low SES**	**Mid-high SES**	**High SES**	**Very high SES**
**Highest educational level achieved** ^**a**^	
No education/elementary/basic dutch (for foreigners)	1.0%	2.1%	–	5.0%	–	–	–	–	–
Primary/basic preparatory vocational/lower vocational	5.8%	12.0%	–	24.0%	–	5.9%	–	–	–
General preparatory vocational^b^/lower secondary or lower college-preparatory/special preparatory	9.7%	20.1%	–	26.1%	29.0%	7.3%	0.1%	0.1%	–
Higher vocational or old vocational classification	27.7%	49.3%	7.9%	44.8%	61.0%	46.8%	26.3%	3.2%	–
Upper secondary or upper college-preparatory	7.4%	3.5%	10.9%	–	10.0%	3.4%	13.3%	14.9%	1.4%
University-level up to bachelor's equivalent	35.4%	13.0%	56.0%	–	–	36.6%	51.0%	64.4%	44.8%
Master's or doctoral/post-graduate	13.1%	–	25.1%	–	–	–	9.3%	17.4%	53.8%
**Gross household income per year**	
<€25,000	22.9%	45.3%	2.2%	72.5%	10.0%	36.6%	9.3%	–	–
Between €25,001 and 35,000	20.1%	23.9%	16.7%	20.8%	61.0%	3.4%	51.0%	9.0%	–
Between €35,001 and 50,000	24.4%	26.0%	22.8%	6.6%	29.0%	46.8%	13.3%	39.6%	–
Between €50,001 and 70,000	19.8%	4.1%	34.2%	–	–	11.5%	26.3%	42.3%	26.1%
Between €70,001 and 100,000	9.8%	0.6%	18.3%	–	–	1.7%	0.1%	8.9%	52.2%
Between €100,001 and 250,000	2.9%	–	5.5%	–	–	–	–	0.1%	20.4%
More than €250,000	0.2%	–	0.4%	–	–	–	–	–	1.3%
Number of respondents (= 100%)	772	369	403	153	85	131	96	200	107

General descriptives of the respondents' highest educational level and gross household income per year as categorized to the different SES groups classifications. ^a^In the Netherlands, secondary education is subdivided into multiple programmes that are oriented toward the needs of the student and include vocational variants. ^b^Corresponds with C-level GCSEs in the UK and 10th grade in the US.

The distinction between the low and high SES groups in the two-SES groups situation was based on whether the individual SES score was lower or higher than zero. This resulted in 369 respondents in the low SES group and 403 respondents in the high SES group. In the six-SES groups situation the cut-off points were defined as follows (see also Figure 2):

^*^SES score −2.41 to −1.13 SES very low

^*^SES score −0.77 to −0.71 SES low

^*^SES score −0.695 to −0.25 SES mid-low

^*^SES score −0.05 to 0.153 SES mid-high

^*^SES score 0.48–1.00 SES high

^*^SES score 1.35–2.66 SES very high.

**Figure 3:**
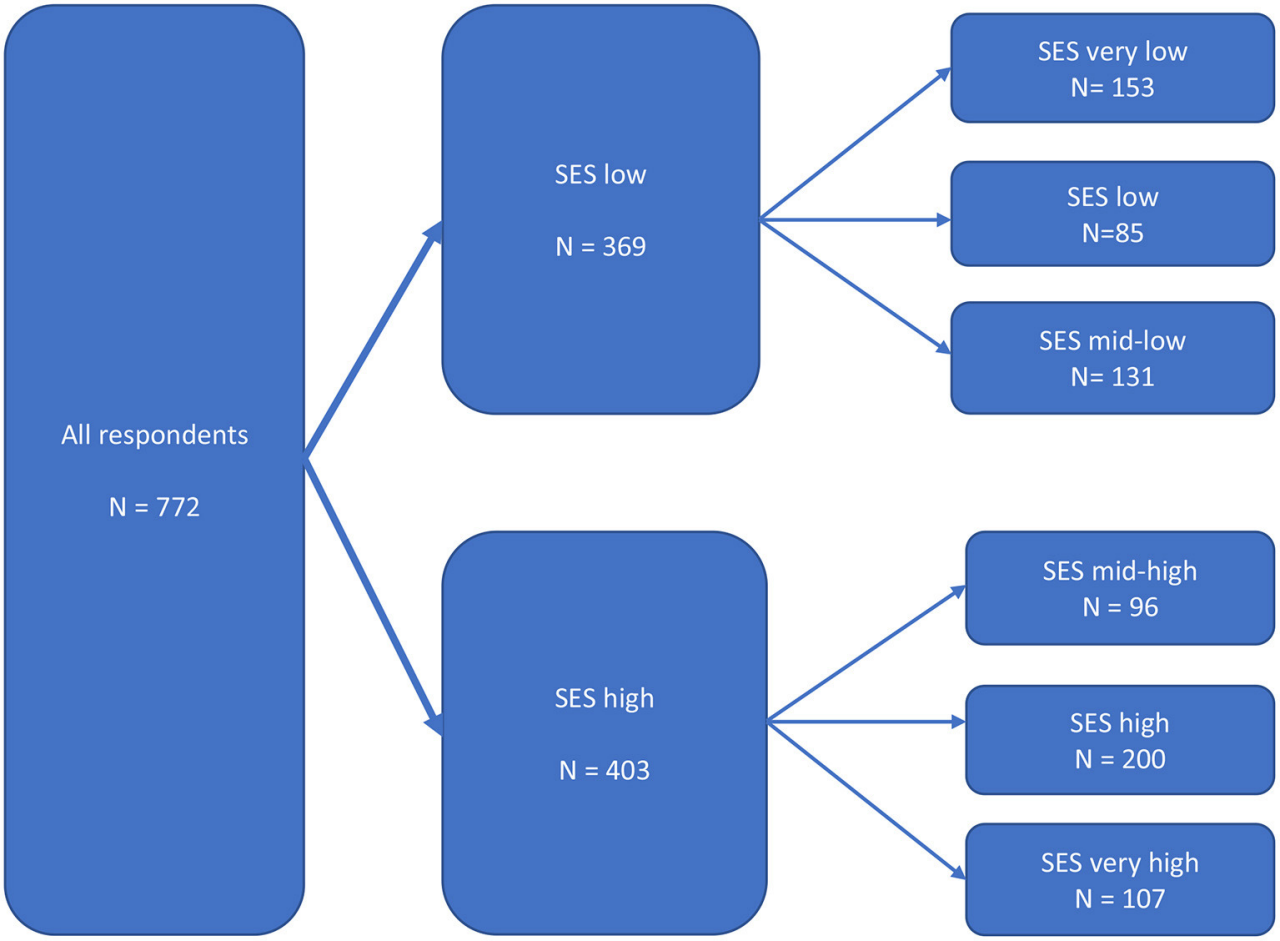
Structure of the empirical analysis.

The six-SES groups situation relates to the two-SES groups situation in a rather simple way: the three groups with the lowest SES scores in the six-group situation belong to the low SES situation, whereas the three groups with the highest SES scores in the six-group situation belong to the high SES situation (Figure 3).

## Results

### Research population

Respondents' average age was almost 49 years (range 18–93). The sample consisted of 48% women and 52% men; 45% had vocational education, 7% secondary education and 48% university education as highest educational level (see Tables 1, 2). Table 1 reveals that the relationship between education and income is not linear, given the combinations of low income/high education and high income/low education as suggested by Flinterman et al. (52).

**Table 2:** Sample and SES groups' socioeconomic and demographic descriptives.

		**Two SES groups** ^**2**^		**Six SES groups** ^**3**^					
	**Total sample**	**Low SES**	**High SES**	**Very low SES (a)**	**Low SES (b)**	**Mid-low SES (c)**	**Mid-high SES (d)**	**High SES (e)**	**Very high SES (f)**
**Type of housing/dwelling**	
Home owner	62.2%	43.8%^‡^	79.0%	30.1%^bcdef^	55.6%^aef^	52.1%^aef^	56.8%^aef^	81.9%^abcd^	93.5%^abcd^
Home renter	37.8%	56.2%^‡^	21.0%	69.9%	44.4%	47.9%	43.2%	18.1%	6.5%
**Age**	
Mean age in years	48.9	51.5^‡^	46.5	55.3^cdef^	54.3^cdef^	45.1^ab^	46.5^ab^	46.1^ab^	47.2^ab^
**Has difficulties meeting financial obligations**	
Mean score^4^	3.74	3.34^‡^	4.11	3.19^cdef^	3.31^def^	3.54^aef^	3.83^abf^	4.08^abcf^	4.40^abcde^
**Household size**	
Total number of people in household (mean)	2.33	2.16^‡^	2.49	1.68^cdef^	2.07^c^	2.77^ab^	2.33^a^	2.54^a^	2.53^a^
**Labour market status** ^**1**^	
Has a paid job	52.0%	36.4%^‡^	66.3%	24.4%^cdef^	38.4%^ef^	49.0%^aef^	57.1%^a^	66.2%^abc^	74.7%^abc^
Retired	18.9%	23.1%	15.0%	33.7%	26.2%	8.8%	20.8%	14.0%	11.7%
Unable to work, incapacitated or chronically ill	10.8%	17.0%	5.1%	21.0%	14.5%	14.0%	9.1%	5.9%	0.1%
Unemployed	6.8%	9.8%	4.1%	13.7%	7.6%	6.7%	4.3%	4.1%	3.9%
Homemaker	4.5%	6.7%	2.6%	4.2%	9.5%	7.6%	3.0%	3.0%	1.5%
Has education and does not work	2.7%	2.6%	2.8%	0.8%	0.1%	6.3%	4.4%	2.8%	1.2%
Miscellaneous	4.3%	4.4%	4.1%	2.2%	3.5%	7.6%	1.3%	4.0%	6.9%
**Gender**	
Male	52.3%	43.7%^‡^	60.1%	39.6%^ef^	38.0%^ef^	52.1%^f^	49.3%^f^	58.3%^ab^	73.2%^abcd^
Female	47.7%	56.3%^‡^	39.9%	60.4%	62.0%	47.9%	50.7%	41.7%	26.8%
Number of respondents (= 100%)	772	369	403	153	85	131	96	200	107

General descriptives of the respondents' type of housing, age, difficulty in meeting their financial obligations and household size as categorized to the different SES groups classifications. ^1^In the Regression Analysis This Variable Was Transformed Into a Dummy Factor (Having a Paid job or not). Statistical Differences Only Relate to This Dummy Factor. ^2^In the Columns of the two SES Groups, Statistical Significance Is Based on *T*-Tests With ‡*p* = 0.000. ^3^In the Columns of the six SES Groups the Superscripts ^a, b, c, d, e, f^Refer to the Statistically Significant Differences (*p* < 0.05) Between the Value in one SES Group and the Values in any of the Other five SES Groups. Statistical Differences Are Based on one-Way ANOVA. ^4^A Higher Mean Score Means Fewer Difficulties Meeting Financial Obligations.

### General overview of the results on health

As illustrated in the model in Figure 3, the analysis was done at three different levels: (1) all respondents, (2) two SES groups, and (3) six SES groups. We will follow the structure of this model when presenting our results.

In general, all respondents perceived their TGH as quite good: more than two-thirds (67.5%) evaluated their TGH as good and/or excellent, in the two-SES groups situation 57.2% for the low SES group and 77.0% for the high SES group. This TGH score was constantly increasing in the six-SES groups situation: from 49.9% in the very low group *via* 58.8% (low), 64.7% (mid-low), 69.8% (mid-high) and 73.2% (high) to 90.3% in the very high group.

When distinguishing between SES groups, average TGH and mean of all the dimensions of health were perceived as better with increasing SES scores while standard deviations decreased (see Table 3). This smaller standard deviation implies that people within a higher SES group have a more common perception of their health and are a rather homogeneous group in this respect. There was quite some heterogeneity in the perceived health of lower SES participants, as SDs within each of the lower SES groups were quite large.

**Table 3:** Evaluation of health and its dimensions in total group, two and six SES groups.

		**Two SES groups** ^**1**^				**Six SES groups** ^**2**^											
	**Total sample**		**Low SES**		**High SES**		**Very low SES (a)**		**Low SES (b)**		**Mid-low SES (c)**		**Mid-high SES (d)**		**High SES (e)**		**Very high SES (f)**	
	**Mean**	**SD**	**Mean**	**SD**	**Mean**	**SD**	**Mean**	**SD**	**Mean**	**SD**	**Mean**	**SD**	**Mean**	**SD**	**Mean**	**SD**	**Mean**	**SD**
Total general health	3.64	0.88	3.41^‡^	0.97	3.86	0.73	3.34^ef^	0.95	3.47^ef^	0.93	3.45^ef^	1.01	3.59^f^	0.79	3.83^abcf^	0.72	4.15^abcde^	0.57
Bodily functions	3.58	1.00	3.32^‡^	1.08	3.81	0.87	3.20^def^	1.11	3.45^f^	1.02	3.38^ef^	1.07	3.64^af^	0.87	3.72^acf^	0.90	4.11^abcde^	0.76
Daily functioning	3.89	0.88	3.67^‡^	0.97	4.09	0.73	3.62^ef^	1.01	3.65^ef^	0.94	3.75^ef^	0.95	3.81^f^	0.75	4.07^abcf^	0.74	4.39^abcde^	0.58
Quality of life	3.75	0.82	3.56^‡^	0.92	3.92	0.67	3.49^ef^	0.86	3.64^f^	0.90	3.59^ef^	0.99	3.70^f^	0.62	3.89^acf^	0.69	4.18^abcde^	0.60
Social and societal participation	3.60	0.86	3.45^‡^	0.92	3.75	0.78	3.39^ef^	0.90	3.48^f^	0.88	3.49^f^	0.96	3.61^f^	0.67	3.66^af^	0.86	4.05^abcde^	0.59
Mental functions and perception	3.86	0.93	3.67^‡^	1.05	4.03	0.76	3.58^ef^	1.09	3.81^f^	0.80	3.69^ef^	1.14	3.74^f^	0.84	4.01^ac^	0.77	4.31^abcd^	0.55
Meaningfulness	3.49	0.54	3.36^†^	1.01	3.61	0.85	3.35^f^	1.05	3.40^f^	0.96	3.34^f^	1.02	3.40^f^	0.89	3.57	0.85	3.86^abcd^	0.75
Number of respondents	772		369		403		153		85		131		96		200		107	

Self Rating of total general health and the six health dimensions by respondents as categorized to the different SES-groups. ^1^In the Columns of the two SES Groups, Statistical Significance Is Based on *T*-Tests With ^†^*p* < 0.001; ^‡^*p* = 0.000. ^2^In the Columns of the six SES Groups the Superscripts a, b, c, d, e and f Refer to the Statistically Significant Differences (*p* < 0.05) Between the Value in one SES Group and the Values in any of the Other five SES Groups. Statistical Differences Are Based on one-Way ANOVA.

**Figure 4:**
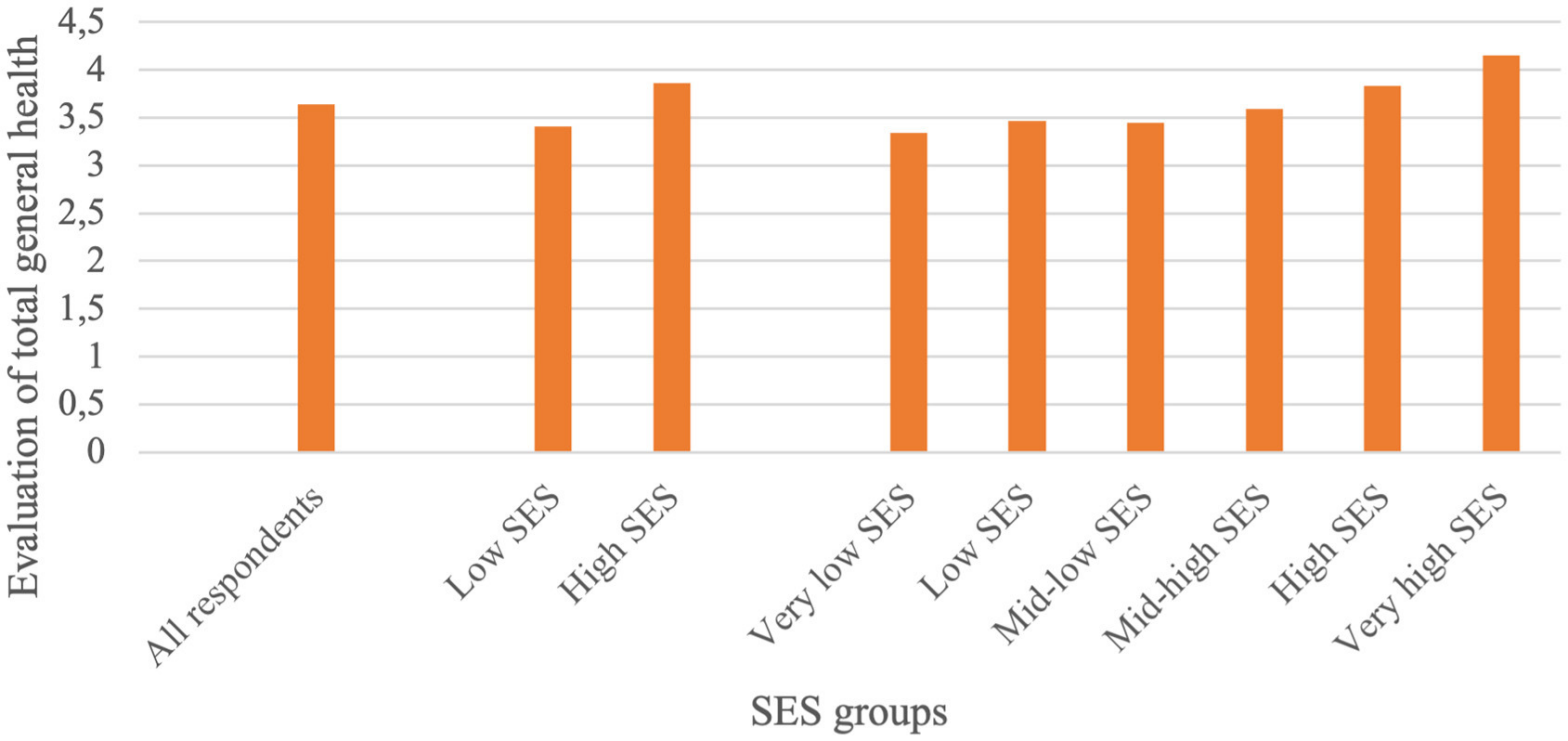
Mean scores of the evaluation of TGH.

Analysing the evaluations of TGH in the different SES groups, Figure 4 summarises the results for the mean TGH scores per SES group. The inequality in the evaluation of TGH is clear. In the two-SES groups situation, TGH was significantly lower in the low SES group than in the high SES group. To increase readability of the results, in this section we will report only the findings that were significant.

In the six-SES groups situation, self-evaluation of health shows a gradient in the pattern of better health with increasing SES. TGH of the four lowest SES groups was the same, yet significantly lower than that of the two highest SES groups. This implies the possible gradual yet critical turning point at which a higher SES has a significant impact on (better) TGH. That turning point did not lie exactly between the low and high SES groups (in the two-SES groups situation), but is part of the (dichotomous) high SES group.

**Figure 5:**
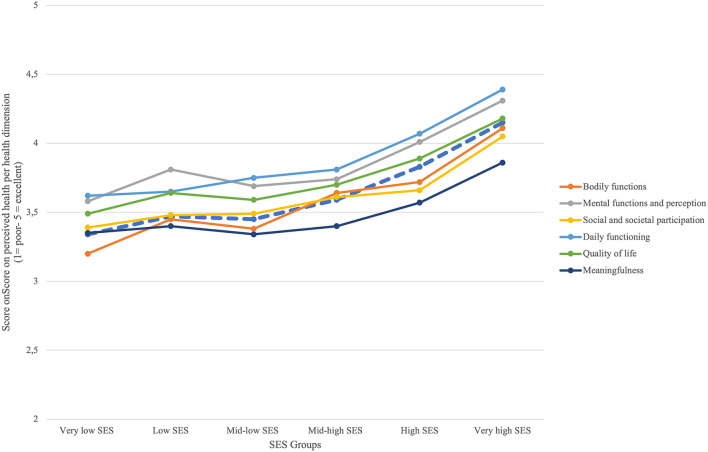
Score on perceived health per health dimension.

A similar gradient was found for each of the six health dimensions (Figure 5; Table 3). These evaluations were significantly higher in the two highest SES groups than in the four lower SES groups. In all dimensions, the lowest SES group and the highest SES group scored significantly lowest and highest, respectively.

Considering the socioeconomic and demographic determinants associated with TGH, respondents in higher SES groups were significantly more likely to own a house, be employed, have larger households, and have less trouble meeting financial obligations (Table 3). Gender was almost equally distributed among total respondents but showed a gradient of more males with increasing SES. When distinguishing between both two-SES and six-SES groups, gender distribution shifted toward significantly higher numbers of males. Age seemed to decrease with increasing SES. The mid-high SES group resembled the three lower SES groups more than the two higher SES groups.

### Results on the relationship between SES and health

When performing a single regression on the impact of the SES score on TGH for all 772 respondents, 10.1% of the variance in TGH was explained by the SES score (standardised β = 0.320; *R*^2^ = 0.101, *p* = 0.000). This shows that the higher the SES score, the higher the TGH is evaluated, and illustrates that the well-known positive impact of SES on health is also present in our data. Given this 10.1% explained variance, other variables could be added to increase it. To this end, in a multiple regression analysis we included the six health determinants, the six socioeconomic and demographic determinants from our model, and participants' individual SES scores. This model is statistically significant (adjusted *R*^2^ = 0.777, *p* = 0.000; see first column in Table 4). However, SES does not have an impact on the evaluation of TGH in this multiple regression (standardised β = −0.025, *p* = 0.244).

**Table 4:** Multiple regression analyses explaining total general health.

	**Total sample**		**Two groups**		**Six groups**					
**Evaluation of**	**Total incl. SES variable**	**Total excl. SES variable**	**Low SES**	**High SES**	**Very low SES**	**Low SES**	**Mid-low SES**	**Mid-high SES**	**High SES**	**Very high SES**
Bodily functions	**0.382 (0.000)**	**0.380 (0.000)**	**0.333 (0.000)**	**0.458 (0.000)**	**0.261 (0.001)**	**0.489 (0.001)**	**0.378 (0.000)**	**0.273 (0.000)**	**0.490 (0.000)**	**0.401 (0.000)**
Daily functioning	**0.292 (0.000)**	**0.289 (0.000)**	**0.352 (0.000)**	**0.219 (0.000)**	**0.330 (0.000)**	**0.432 (0.000)**	**0.367 (0.000)**	**0.307 (0.000)**	**0.151 (0.004)**	**0.521 (0.000)**
Quality of life	**0.261 (0.000)**	**0.263 (0.000)**	**0.300 (0.000)**	**0.182 (0.000)**	**0.383 (0.000)**	0.126 (0.153)	**0.219 (0.000)**	**0.370 (0.000)**	**0.211 (0.000)**	−0.102 (0.177)
Social and societal participation	**0.065 (0.007)**	**0.066 (0.006)**	0.032 (0.343)	**0.092 (0.008)**	0.013 (0.814)	−0.010 (0.868)	0.078 (0.198)	−0.115 (0.061)	**0.162 (0.002)**	0.094 (0.169)
Mental functions and perception	−0.024 (0.334)	−0.025 (0.322)	−0.064 (0.080)	**0.110 (0.002)**	−0.091 (0.152)	0.020 (0.737)	−0.079 (0.198)	0.076 (0.221)	0.077 (0.151)	**0.198 (0.002)**
Meaningfulness	−0.021 (0.292)	−0.022 (0.270)	−0.042 (0.124)	−0.014 (0.639)	**−0.151 (0.004)**	−0.076 (0.197)	0.009 (0.825)	−0.052 (0.266)	−0.022 (0.609)	0.051 (0.429)
**Socioeconomic determinants**	
SES	−0.025 (0.244)				
Home ownership or renting^a^	**−0.084 (0.000)**	**−0.077 (0.000)**	**−0.112 (0.000)**	**−0.059 (0.024)**	**−0.216 (0.000)**	**−0.112 (0.039)**	−0.076 (0.068)	0.039 (0.388)	**−0.098 (0.009)**	0.001 (0.982)
Age	**−0.058 (0.005)**	**−0.055 (0.007)**	**−0.082 (0.006)**	−0.010 (0.732)	**−0.129 (0.012)**	−0.032 (0.618)	−0.036 (0.426)	−0.045 (0.342)	**−0.090 (0.032)**	**0.197 (0.003)**
Difficulties meeting financial obligations	**0.052 (0.011)**	**0.045 (0.021)**	0.053 (0.056)	0.019 (0.478)	0.031 (0.537)	0.081 (0.099)	**0.095 (0.034)**	**0.130 (0.005)**	−0.008 (0.827)	−0.075 (0.190)
Household size	−0.018 (0.321)	−0.021 (0.260)	−0.033 (0.230)	−0.008 (0.747)	**−0.106 (0.018)**	0.039 (0.437)	0.034 (0.426)	0.006 (0.906)	**−0.087 (0.016)**	**0.174 (0.004)**
Labour market status: employed or not^b^	−0.012 (0.555)	−0.015 (0.446)	**−0.080 (0.005)**	**0.083 (0.003)**	−0.043 (0.413)	−0.086 (0.151)	**−0.089 (0.026)**	**0.218 (0.000)**	0.049 (0.209)	0.044 (0.508)
Gender	0.002 (0.902)	0.005 (0.795)	−0.026 (0.305)	**0.067 (0.012)**	−0.045 (0.348)	**0.159 (0.005)**	−0.051 (0.172)	**0.100 (0.027)**	0.041 (0.295)	−0.009 (0.869)
Adjusted *R*^2^	0.777	0.776	0.785	0.758	0.726	0.852	0.856	0.868	0.763	0.730
Significance of the model	**0.000**	**0.000**	**0.000**	**0.000**	**0.000**	**0.000**	**0.000**	**0.000**	**0.000**	**0.000**
*F*-value	207.282	224.337	22.718	13.511	34.573	41.449	65.389	53.012	54.442	24.887
Degrees of freedom	13	12	12	12	12		12	12		12

Data in the table represent regression coefficients of six health dimensions and the sociodemographic determinants explaining self-rated total general health. Significance level in parentheses and values are in bold. ^a^ A Minus Sign Means a Negative Impact on TGH of Renting a House (vs, a Positive Impact of on TGH of Owning a House). ^b^ A Minus Sign Means a Negative Impact on TGH of not Having a job (vs, a Positive Impact of on TGH of Having a job).

The disappearing impact of SES might be due to the fact that the influence of SES on the perception of TGH is “taken over” by other variables in the model. However, SES may still affect TGH one way or another given the significant simple regression's output. As a next step in our analysis, we therefore focus on the possible relationships between the six health determinants together with the six socioeconomic and demographic determinants in explaining TGH in the entire sample as well as within a specific SES group. We performed multiple linear regression analyses both for the traditional two-SES groups and for each of the newly developed six-SES groups. The results of our analyses are presented in detail below.

### Factors determining all respondents' evaluation of TGH

When analysing the data of all respondents without the SES variable, the perception of TGH was explained by four of the six health determinants and by three of the six socioeconomic and demographic determinants (second column in Table 4). Here a higher score on perceived TGH could be largely explained by a higher score on the evaluation of bodily function and daily functioning, followed by a higher score on evaluation of quality of life. Additionally, but to a lesser extent, people reporting owning a house, people scoring higher on social and societal participation, younger people, and those with no or fewer difficulties meeting financial obligations scored higher on their perceived TGH. Perceived TGH was however not influenced by evaluation of the other two health dimensions of meaningfulness and mental function, or by the socioeconomic and demographic variables of gender, household size and labour market status because no significant standardised β's were found.

### Factors determining the evaluation of TGH by distinguishing between two groups of SES

When distinguishing between two groups of SES, multiple regression analyses of both SES groups (Table 4) show that scores on bodily functions, daily functioning and quality of life have a large positive impact on perceived TGH in both SES groups. However, given the size of the standardised β's the positive impact of each of these three health determinants differs: the positive impact of bodily functions was larger in the high SES group, while that of daily functioning and quality of life was larger in the low SES group. In both SES groups home owners perceived better TGH than renters, while the impact of the housing situation was larger in the low SES than in the high SES group.

The low and high SES groups differ on the impact of the health dimensions of mental functions and social-societal participation and the socio-demographic variables of gender, age and labour market status. These two health dimensions impacted TGH positively only in the high SES group. Also, in the high SES group only, women perceived better TGH than men, whereas age only impacted perceived TGH in the low SES group, showing decreasing health with increasing age. Lastly, having a job increased perceived health in the low SES group but decreased it in the high SES group. The health determinant of meaningfulness and the socioeconomic and demographic determinants of household size and difficulties meeting financial obligations did not impact the perception of TGH in either of these two SES groups.

In sum, with respect to the health dimensions determining the evaluation of TGH by distinguishing between two groups of SES, both SES groups show similarities as well as differences. In both groups TGH was largely and positively affected by three health dimensions: bodily functions, daily functioning and quality of life. TGH was positively affected by evaluation of social-societal participation and mental function only in the highest SES group. In both SES groups home owners perceived better health than renters. Having a job, on the other hand, impacted TGH positively in the low SES groups but negatively in the high SES group.

### Factors determining the evaluation of TGH by distinguishing between six groups of SES

In all six SES groups the health dimensions of bodily functions and daily functioning contributed positively to perceived TGH. The perception of the quality-of-life dimension impacted TGH in four out of six SES groups—not in the low SES and not in the very high SES group. The size of the standardised β however reveals that this impact differs per SES group. In the very low SES group, perceived quality of life had the biggest impact on TGH from two perspectives: compared to all other significant impacts in this SES group and compared to the significance of this quality-of-life dimension in all other SES groups. The dimension of mental functioning contributed positively only to the very high SES group's TGH. Evaluation of the meaningfulness dimension impacted on perceived TGH only in the very low SES group: respondents scoring higher in meaningfulness perceived lower TGH. The dimension of social and societal participation explained only perceived TGH in the high SES group.

None of the socioeconomic and demographic determinants impacted TGH in all six SES groups. Home ownership contributed positively to perceived TGH in the two lowest SES groups and the high SES group. The younger people are and the smaller the households, the better they perceived their TGH in the very low and the high SES groups, whereas the opposite was found in the very high SES group. Only the mid-low and the mid-high SES groups perceived that TGH was affected positively by having fewer difficulties meeting financial obligations. However, having a paid job contributed positively to perceived TGH in the mid-low SES group but negatively in the mid-high SES group. Lastly, women perceived better TGH only in the low SES and mid-high SES groups but not in the other SES groups.

In sum, these results show that different health dimensions and different socioeconomic and demographic dimensions determine the perception of TGH differently per SES group when distinguishing between six groups of SES. All six SES groups had two health dimensions (out of the six) in common that positively determined their TGH: bodily functions and daily functioning. None of the six socioeconomic and demographic variables included in this study impacted TGH in all SES groups—some variables impacted TGH positively in one SES group and negatively in another: for instance, having a paid job had a positive effect on TGH in the mid-low SES group but a negative effect in the mid-high group.

## Discussion

This paper focused on three research questions, all related to the complex relationship between socioeconomic status and perceived total general health. Most respondents perceived their health quite positively: a little more than two-thirds evaluated their TGH as good and/or excellent. The lower standard deviation in TGH scores of respondents within a higher SES group shows a more common perception of their health; they are quite a homogeneous group in this respect. By contrast, there was a wide difference in perception of TGH between respondents within the lower SES groups, as shown by their higher standard deviation. In general, respondents in higher SES groups apparently not only perceived better TGH but also shared a more common evaluation of their health.

### Answering the research questions

The first research question was about which health dimensions in life determine the perception of TGH. Using the concept of positive health, it appears that four out of the six dimensions impact the evaluation of TGH. The evaluation of bodily functions, daily functioning, quality of life, and social and societal participation had a positive impact on the evaluation of TGH, and these are also the most important health dimensions when determining TGH.

All SES groups had in common the positive impact of bodily functions and daily functioning on their TGH. However, the magnitude of the impact of these two dimensions as well as of the other two dimensions (as measured *via* the standardised β's in our regressions) differed per SES group. The impact of the two health dimensions on mental functioning and meaningfulness seemed to be rather small and only present in a few SES groups. This is in line with findings of Stronks et al. (70) showing in a concept map where, regardless of educational level, on the one hand aspects like “absence of disease and functioning” and “health-related behaviours” and on the other hand aspects like “social life” and “attitude toward life” were perceived as important characteristics of health.

Evaluation of the other two dimensions (mental functions and meaningfulness) did not impact all respondents' TGH. It could be argued that people may not be fully aware of the possible impact of these two dimensions on perceived TGH as separate dimensions. This interpretation is in line with Vogel et al. (71) observing that mental health and meaningfulness have a close connection influencing self-reported health (SRH) and consequently are undistinguishable by people. Another interpretation could be that people only become aware of the importance of these dimensions in specific situations. This means more aware of the dimension meaningfulness when perceiving illness, as is the case only in the very low SES group where meaningfulness negatively impacts on TGH. However, more research on this specific topic is needed, given the inconclusive results in this respect. For, in the literature on the topic of SES and health in relation to meaningfulness there are no consistent findings with replicated outcomes. To illustrate, Joan and Reutter (72) showed that increasing income increases SRH in Canadian women with the sense of coherence (consisting of meaningfulness, comprehensibility and manageability) as intervening variable. Vogel et al. (71) found that meaningful activities increased SRH in incarcerated older men and Steptoe and Fancourt (73) report that SRH is an important factor for living a meaningful life whereas the reverse relationship is not that clear. The different indicators used for meaningfulness and evaluating TGH could explain these different outcomes. Standardising of methods and approaches would benefit gaining more insight in the impact of meaningfulness on TGH.

Concerning the dimension mental functions and perceptions impacting TGH only in the very high SES group might imply that a certain level of education and income is needed for this impact to occur. This would be in line with findings of Kim and Cho (74) that especially high SES are burdened with work-life conflict decreasing mental health. These people do realize the importance of mental functions and perceptions once they have demanding high income positions; they then do realize that there is more in life than only a high income earning job. Furthermore, on the positive side, higher SES groups have been shown to report better mental health (75).

The second research question was about which socioeconomic and demographic variables determine the perception of TGH. It turned out that type of housing, age, and difficulties meeting financial obligations impacted on all respondents' perceived TGH, which proportionately worsens with increasing home renting, age, and difficulties meeting financial obligations. Gender, household size and labour market status (= having a job or not) did not impact all respondents' TGH.

These determinants of perceived TGH are in line with earlier findings on determinants of objective health. First, it is established that objective health decreases and use of healthcare increases with age (76, 77). Second, it is widely known that housing conditions are a determinant of health. People living in substandard, often rented housing in deprived neighbourhoods have more impaired health than home owners in affluent neighbourhoods (1, 30, 78). Meyer et al. (19) (pp1734) formulated in the abstract their results in a somewhat broader context of the impact of the deprived neighbourhood instead of the bad housing situation: “Low SES was associated with greater neighborhood safety concerns, which were negatively associated with physical activity, which was then negatively related to mental health and SRH.” Third, having trouble meeting financial obligations is at the core of socioeconomic inequality in both objective and self-rated health (29, 79, 80).

Concerning gender differences in health, the present study shows no differences in perceived TGH between men and women. However, since men tend to show more risky health behaviour and are more predisposed to suffer significantly more from chronic diseases than women (21, 35), they might have been expected to perceive lower TGH than women. As we did not check for risky behaviour or chronic diseases, it is impossible to directly relate these findings to our results on perceived TGH. It might be speculated that good TGH can be perceived despite having a chronic disease, since other dimensions of health like daily functioning or quality of life might compensate for the impaired bodily function caused by the disease. It might also be that people accept their situation and adapt to it as is illustrated by the observation that older people compare their own decreased health with age peers who have even worse health. Concerning risky behaviour, it might be speculated that men do not perceive their behaviour as risky but more as a socially subjective norm which therefore does not influence their perception of TGH.

The determinant of household size not affecting perceived health confirms the results of a study suggesting a more social, hereditary component of SES negatively impacting health rather than family size (39). However, effect of family size on objective or self-rated health is inconclusive. Some studies find positive effects of larger families because of mothers staying at home to care for the children and hence exerting more parental control (42) and reduced obesity in families with more siblings (40). Others report negative effects of family size on health, as larger families show increased household chaos, which causes maternal stress (41, 43), plus overcrowding in early life leads to increased risk of multimorbidity in midlife (44).

Lastly, the determinant of labour market status might have been expected to affect perceived TGH, as unemployment or poor job satisfaction have detrimental effects on health (36–38). Our findings do not corroborate this expectation. However, being unemployed in general means less income and thus a higher likelihood of having difficulties meeting financial obligations. Hence a possible explanation for perceived TGH being unaffected by the determinant of labour market status is that the determinant of having trouble meeting financial obligations is compensating for that. Besides, having a job is not a guarantee for health as such, but adequate payment for a job is (1, 22).

The third research question was about the extent to which the relationships between TGH, health determinants, and socioeconomic and demographic determinants differ between various groups of SES, distinguishing six instead of the traditional two groups of high and low SES. Since discussing all the findings would complicate the readability, we will focus on only the signifcant findings.Our six-SES groups approach provided more detailed information than the traditional two-SES groups approach. It also produced more refined information on the similarities and differences between the SES groups. Similarities between all six groups could be found for impact of two health determinants of perceived TGH—bodily functions and daily functioning. The mean perceived TGH score did not differ between the four lowest SES groups, while these differed significantly from the mean perceived TGH score in the two highest SES groups. None of the socioeconomic and demographic determinants impacted perceived TGH in all the SES groups. The impact of all these health, socioeconomic and demographic determinants was contingent upon the specific SES group. There were different gestalts of the health dimensions and the socioeconomic and demographic variables, suggesting that health was perceived differently by each SES group. These findings on subjective SRH evaluation are in line with existing literature on inequality of health defined by professionals in terms of the more objective health indicators: health is evaluated better as people's SES is higher (80, 81). In terms of the methodology applied, the finding that our subjective approach leads to a conclusion similar to the professionals' opinion used thus far is new to the existing literature. This corroborates the findings of Stronks et al. (70) which show differences between three levels of educational groups by conceptualising health using concept maps.

In general, it is important to emphasise that the six-SES groups approach shows there is a gradient instead of a linear pattern in the magnitude of perceived TGH and its six health determinants across the six SES groups. The four lower SES groups (very low, low, mid-low and mid-high) did not differ from each other on perceived TGH score or the score on its six health determinants. However, all of these scores were significantly higher in the two highest SES groups (high and very high) than in the other four SES groups.

A similar three-step gradient seems to be present in the scores on the significant socioeconomic and demographic determinants in the six SES groups, as these determinants impacted perceived TGH the most in the very low SES group, less in the following three SES groups (low, mid-low, mid-high), and little in the high and very high SES groups.

The gradient instead of linear trend in the relationship between SES and health inequality has been reported in several studies (29, 31, 79, 82). By dichotomising SES at a median cut-off point or studying SES determinants separately, possible socioeconomic effects on perceived health might have been obscured. More SES groups than only two should be distinguished, also in order to develop more effective interventions to improve people's health.

The results of our study on the determinants of perceived TGH as a measure of SRH are not only in line with the existing literature, they also add to it on three accounts. First, perceived TGH and hence SRH were operationalised by elaborately scoring on 32 items corresponding not only to the physical and psychological dimensions but also to the social-societal, quality of life, meaningfulness and daily functioning dimensions, adding new items to the scales as used in the SHQOL and SF12 questionnaires (29, 68, 78, 83, 84).

Second, we calculated a six-level SES score based on a newly created individual SES score *via* factor analysis of gross household income and education instead of using a dichotomised SES score of high and low. In this way we corrected for the possibility that during the life course income can rise or fall regardless of educational level. As has been shown, detrimental life events like divorce or unemployment due to crises like the recession of the early 2000 s or the current COVID-19 pandemic (47, 48, 85) can cause serious losses of income for both the higher and the lower educated. It is even speculated that the impact of these losses in income affect the total wellbeing of the low SES groups more than that of the high SES groups (47, 48). In addition, the impact of meaningfulness on TGH in the very low SES group was obscured when only distinguishing two SES groups. Therefore, in future studies distinguishing between more than 2 groups is recommended.

Third, we evidenced a non-linear gradient in SES impacting perceived TGH and its six health determinants. With regard to possible health-promoting interventions to improve health or TGH and reduce the socioeconomic gap in health, our findings support the suggestion made by Stronks et al. (70) (pp. 8) that “the way health is conceptualized, challenges the legitimacy of policies that are based on a notion of health that resonates the conceptions that are valued in higher socioeconomic groups….” Translated from policies to health-promoting interventions, this means that health-promoting interventions should be tailored to the way health is perceived and valued by the target population. More specifically, such customisation should be oriented toward the importance and magnitude of the six health dimensions perceived by the specific SES group being targeted. This topic, which refers to a typical marketing approach, will be elaborated upon below.

### Implications of the significant relationships between TGH and the six health determinants in the six SES groups

**Figure 6:**
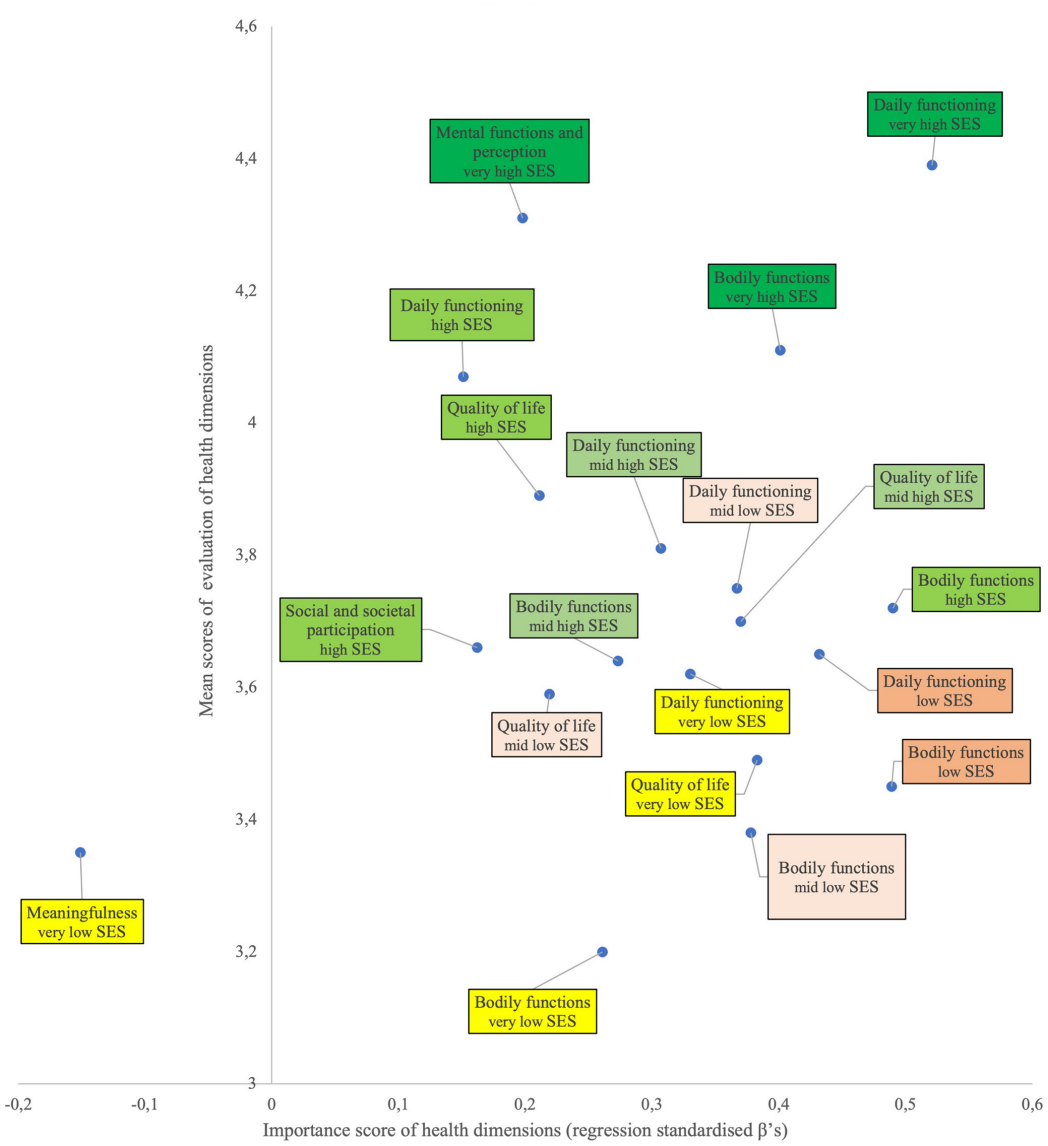
Significant importance and mean scores per health dimension for all six SES groups. The relation between the standardised β's of the regression analysis as a measure for significance of impact (importance score; horizontal axis) and the mean score of the evaluation of each of the six health dimensions (vertical axis) per SES group. A higher score on the horizontal axis means bigger impact on the health dimension in the SES-group. A higher score on the vertical axis means a higher mean score of the health dimension in the SES group. The position in the graph is a measure for the importance of a health dimension and the way this dimension is valued by a person in a SES group.

From marketing literature, it is known that the combination of mean scores and importance is critical in making decisions and setting priorities about which changes should be made in marketing strategies, for instance to better meet customer needs (86). In this analogy, combining the *significant impact* of the evaluation of each of the six health dimensions on TGH in each SES group from Table 4 (importance scores based on standardised β's) with how high or low the evaluations are in each of the six SES groups from Table 3 (mean scores) yields the basis for setting priorities in potential interventions (Figure 6). For, priorities can be based on the notion that the highest priorities for interventions should be directed to those health dimensions that are considered as important but have a low evaluation score. Health dimensions with a high evaluation score and a high importance score should remain at that level and do not need interventions to improve this situation. It remains to be seen whether interventions are needed for those health dimensions that have a low importance score when financial means are scarce.

Health inequality is shown by the positioning of the very high SES group in the upper right corner of the graph. The other SES groups are positioned at lower spots in the graph, moving in roughly descending order to the lower left of the graph with the very low SES group. Some remarkable patterns do stand out. First, the importance score for evaluating the significant health dimensions in the mid-high and high SES groups is rather low (i.e., placed more to the left of the graph) compared to the scores in the other lower SES groups. This indicates that health on these determinants is perceived the same but rated as more important by the lower SES groups. Health-improving interventions aimed at daily functioning would therefore be more effective in the lowest three groups, whereas interventions aimed at bodily functions would yield a higher effect in the low and high SES groups.

Second, other significant dimensions are placed more to the upper-left portion of the graph, indicating a smaller importance but still a rather high score on the evaluation itself. Determinants in this part are perceived as good-very good and of low/lower importance, meaning that interventions aimed at improving these determinants will have little to no effect in these SES groups.

Third, the middle portion of the graph shows a scattered pattern for the very low, low, mid-low and mid-high SES groups (it has been shown that these four—lower—SES groups are quite similar and differ significantly from the two higher SES groups). This indicates that perceived TGH was determined by different gestalts of the evaluation of the health dimensions and their importance to respondents from these four SES groups. These gestalts were different from those in the two higher SES groups. When aiming to reduce socioeconomic differences, mixed interventions targeting the determinants of bodily functions, daily functioning and quality of life would be indicated. The higher health-promoting effectiveness of implementing an intervention mix has been shown in a study stimulating physical activity in prevocational secondary education (87).

Lastly, meaningfulness in the very low SES group was the only dimension with a negative impact on TGH, while the importance score was the lowest of all evaluations. As mentioned previously, this might suggest that people become aware of the psychological dimension of meaningfulness only when TGH is perceived as low, as was the case in the very low SES group. Further research is needed to gain more insight into this determinant affecting perceived TGH in the very low SES group.

In summary, in all six-SES groups situations investigated the evaluation of daily functioning and bodily functions had a very large and positive impact on the evaluation of TGH. Given their high-importance score, these dimensions are the ones most determining equality or inequality in health. Also, quality of life often plays an important role. It appears that the evaluation of some health dimensions (i.e., mental functions and meaningfulness) did not have an impact on TGH in this sample. It may be that participants did not (yet) realise that these dimensions are also relevant in determining TGH or only start realising their importance when they are/become ill. This study shows that different SES groups perceive different health determinants as important to their health, so there is no one-size-fits-all intervention. This could be the starting point for two approaches: to raise awareness in the SES groups of the importance of the other determinants participants do not (yet) perceive as important, and to implement health-promoting interventions matching the perceptions of the SES group. From a salutogenic and capability perspective, the latter might be preferred because first these approaches are focused on stimulating people to use their health assets; in salutogenesis these are referred to as general and specific resistance resources (88, 89) and in capability terms these are related to conversion factors (90). Second, they have the capability aspects of autonomy and freedom to participate (54, 62, 63) instead of implementing general tailored interventions from a professional's perspective based on top-down analysis of determinants.

The marketing discipline would suggest applying the concept of market segmentation when health-promoting interventions should be SES-group specific. Market segmentation aims to develop several market segments when the total market or population is not homogeneous. Each market segment is supposed to consist of people who are similar to each other in terms of particular needs or problems; they are also supposed to react in a similar way to marketing stimuli, like a particular message or health intervention. That will maximise the effectiveness of the investment. Each segment is homogeneous in itself while there is great heterogeneity between the various segments. It is critical to have an understanding of the behaviour of the people in a particular segment, e.g., an SES group: what do they deem relevant and important (a graph like Figure 6 may be very helpful here), how will they respond to certain interventions, etc. In this way it is not a one-size-fits-all approach which would be implemented but a tailor-made, specific market segment approach.

### Possible limitations and critical reflections

Whereas, our study adds to current conceptions of health and its socioeconomic determinants, especially the importance of discriminating between more than two levels of SES, some critical reflections are in place.

First, as mentioned, there was a slight overrepresentation of higher-educated respondents in the whole sample, which might have affected analysis and results. We were aware of this possible bias and corrected for it by calculating SES scores using a factor analysis diluting this relative educational disbalance. It is therefore unlikely for this disbalance to have affected the analysis and results to any considerable extent.

Second, in our study the group of people with perceived poor health is not that large. A study including only persons with perceived poor health might provide new insights into the importance of the mental functioning and meaningfulness determinants of health for this group of people. It may be that people do not realise how important these two determinants are for their health as long as they feel healthy.

Third, when forming SES groups using factor analysis the number of respondents differed per SES group, and especially the low and mid-high SES groups have fewer respondents than the rest. Although the rule that the number of respondents should be larger than the squared number of independent variables in our regression equations was not met, we do not consider that as a serious flaw given the robustness of the statistical technique of regression analysis. Still, a larger number of respondents in some SES groups would have been preferred.

Fourth, comparing the two-SES groups approach with the six-SES groups approach reveals that the traditional dichotomy is too simple and may lead to ineffective interventions. The six-SES groups approach reveals that the high SES group from the dichotomy contains a subgroup (mid-high) that highly resembles all three SES groups from the traditional low SES group (and these four SES groups could be regarded as one group) but differs significantly from the other two high SES groups. It would however be incorrect to conclude from this that only three new groups of SES should be distinguished when developing interventions (the four lower groups, the high group and the very high group), as the *importance* of the determinants of TGH differs for each of the six SES groups. Since interventions should be focused on the determinants deemed important, we suggest fine-tuning the interventions to as many specific SES groups as narrowly defined as possible to achieve maximum effectiveness. In all six-SES groups interventions may relate to features of bodily functions and daily functioning being important in all groups. Quality-of-life issues are important in four of the six groups, and meaningfulness as well as mental functions in two specific groups. The impact of having a job is important in two groups: the very low (positive impact) and the mid-high (negative impact) groups. These refined insights could only be obtained by applying the concept of positive health in this study on the impact of SES on health and by challenging the traditional notion of a dichotomy in SES groups.

## Data availability statement

The raw data supporting the conclusions of this article will be made available by the authors, without undue reservation.

## Ethics statement

It was judged that approval of a medical ethics committee was not mandatory for this research because participants did not undergo physical examination nor were bodily fluids or other medical data collected. Therefore, the Medical Research Involving Human Subjects Act does not apply to the current study. The Board of Silverbrains approved the research design and protocols for the data collection and analysis. The research was carried out in accordance with the rules and guidelines of the Dutch Market Research Organization (MOA) and the Dutch Policy Research Association (VBO) which are in line with the rules and guidelines of the European Association of Market Research (ESOMAR). The respondents voluntarily participated in the study; they are members of the RMI Internet panel. When RMI started their Internet panel years ago, all respondents agreed to participate in this panel study and written informed consent was obtained from all panel members (they all are 18 years or older). To encourage people to participate in studies, respondents receive a small fee of one euro for every completed questionnaire.

## Author contributions

HK applied his expertise in marketing research, especially on the socioeconomic, demographic determinants of health, developed the questionnaire, collected the data, prepared the figures, and performed regression analysis. JD is an expert in health determinants, the concept of positive health, including theories on health inequalities, and added one-way ANOVA analysis. Both authors wrote and reviewed the manuscript. All authors contributed to the article and approved the submitted version.

## Conflict of interest

The authors declare that the research was conducted in the absence of any commercial or financial relationships that could be construed as a potential conflict of interest.

## Publisher's note

All claims expressed in this article are solely those of the authors and do not necessarily represent those of their affiliated organizations, or those of the publisher, the editors and the reviewers. Any product that may be evaluated in this article, or claim that may be made by its manufacturer, is not guaranteed or endorsed by the publisher.

## Abbreviations

SD: standard deviation
SES: socioeconomic status
SRH: self-rated health
TGH: total general health
WHO: World Health Organization.
